# Quantitative fluorescence spectroscopy and flow cytometry analyses of cell-penetrating peptides internalization pathways: optimization, pitfalls, comparison with mass spectrometry quantification

**DOI:** 10.1038/srep36938

**Published:** 2016-11-14

**Authors:** Françoise Illien, Nicolas Rodriguez, Mehdi Amoura, Alain Joliot, Manjula Pallerla, Sophie Cribier, Fabienne Burlina, Sandrine Sagan

**Affiliations:** 1 Sorbonne Universités, UPMC Univ Paris 06, Ecole Normale Supérieure, CNRS, Laboratoire des Biomolécules (LBM), 4 place Jussieu, 75005 Paris, France; 2 Département de Chimie, Ecole Normale Supérieure, PSL Research University, UPMC Univ Paris 06, CNRS, Laboratoire des Biomolécules (LBM), Paris, France; 3 Center for Interdisciplinary Research in Biology, Collège-de-France, PSL Research University, 11 place M. Berthelot, Paris, France

## Abstract

The mechanism of cell-penetrating peptides entry into cells is unclear, preventing the development of more efficient vectors for biotechnological or therapeutic purposes. Here, we developed a protocol relying on fluorometry to distinguish endocytosis from direct membrane translocation, using Penetratin, TAT and R9. The quantities of internalized CPPs measured by fluorometry in cell lysates converge with those obtained by our previously reported mass spectrometry quantification method. By contrast, flow cytometry quantification faces several limitations due to fluorescence quenching processes that depend on the cell line and occur at peptide/cell ratio >6.10^8^ for CF-Penetratin. The analysis of cellular internalization of a doubly labeled fluorescent and biotinylated Penetratin analogue by the two independent techniques, fluorometry and mass spectrometry, gave consistent results at the quantitative and qualitative levels. Both techniques revealed the use of two alternative translocation and endocytosis pathways, whose relative efficacy depends on cell-surface sugars and peptide concentration. We confirmed that Penetratin translocates at low concentration and uses endocytosis at high μM concentrations. We further demonstrate that the hydrophobic/hydrophilic nature of the *N*-terminal extremity impacts on the internalization efficiency of CPPs. We expect these results and the associated protocols to help unraveling the translocation pathway to the cytosol of cells.

Since their discovery twenty years ago, cell-penetrating peptides (CPPs) or protein transduction domains (PTDs) have been described as promising drug delivery systems. There are increasing numbers of successful applications of CPPs/PTDs *in vivo*^1^,^2^,^3^,^4^,^5^. However, one of the limitations to their wide and diverse application is the diversity of their uptake pathways^6^. The CPP and its cargo may end up free in the cytosol and reach their biological target only following translocation, but remain confined in intracellular vesicles after endocytosis, unless subsequent events such as endosomal rupture occur. Along with the development of CPPs/PTDs as vectors to carry various macromolecules for targeted cellular therapies, engineering new CPPs/PTDs with optimized transport and internalization capacities would greatly benefit from the understanding of cell entry mechanisms that still remain subject of controversy in the field^7^. In particular, one should understand how these peptides cross cell membranes. The translocation pathway differs from endocytosis because it still occurs at low temperature (<12 °C)^8^, although with lower efficiency due to decreases in cell membrane fluidity and dynamics.

The study herein directly addresses the issue of the quantification and the mechanisms of cell entry, by providing reliable and robust methods to detect total CPPs/PTDs inside cells. Measurement of the total amount of internalized species irrespective of its localization, cytosolic or vesicular, is an important task since it would encompass all uptake pathways^9^,^10^,^11^. Indeed, CPP internalization and subsequent intracellular traffic are highly dynamic processes, and quantitative snapshots of CPPs/PTDs internalization in restricted areas of the cells, might only provide an incomplete picture of CPP functionality. Therefore, quantitative measurements of local and total peptide in cells are complementary and both informative on CPP/PTD efficacy.

To study the internalization of CPPs /PTDs, fluorescence-based techniques, such as flow cytometry or fluorescence microscopy imaging are predominant in the litterature^12^. These techniques allow monitoring CPP accumulation and for imaging, its intracellular distribution. These techniques rely on the conjugation of a fluorochrome or a biotin to the studied CPP sequence. Irrespective of the nature of the tag added to track their fate, most CPPs strongly interact with the cell surface generating a pool of membrane-bound peptides usually defined as high membrane- or cell surface binding peptides, which cannot be eliminated by saline washings^12^,^13^,^14^. The cell-surface fraction of these peptides may represent from 10 up to 100 times the quantity of internalized peptides as we previously reported^14^,^15^. Therefore, to measure accurately the amount of intracellular peptides, whatever the protocol and method used, one needs to distinguish the internalized from the cell surface bound peptides.

We had previously developed a reliable and robust protocol coupled with mass spectrometry analysis to quantify separately the amounts of internalized and membrane-bound peptides and to detect the products of peptide intracellular degradation^14^,^16^. When performed at 4–12 °C, a temperature at which all endocytic processes are inhibited, uptake only relies on translocation that could be accurately measured^15^. In the present study, we have included fluorescence spectrocopy or flow cytometry to quantify CPP internalization efficacy. We first tested whether fluorometry and mass spectrometry quantification of intracellular CPPs/PTDs from cell lysates give convergent results. In a second series of experiments, we have extended our comparative analysis to flow cytometry, a widely used technique to compare the efficiencies of CPPs/PTDs. We compared 37 °C and 4 °C conditions to distinguish translocation from endocytosis pathways on different CHO cell lines to evaluate the contribution of cell surface negatively charged glycosaminoglycans and sialic acids for each pathway. These studies were performed on the three most studied CPPs/PTDs, Penetratin (PEN), TAT and R9 (Fig. 1). We chose carboxyfluorescein (CF) to label the peptides because of its wide and general use in biological studies, beyond the field of CPPs/PTDs.

## Results

### Fluorometry and mass spectrometry for absolute quantification of internalized peptides lysis conditions

Fluorescein is classically used to label peptides and proteins in order to follow their distribution in cells by live microscopy imaging. This fluorochrome is solvatochromic, implying that the surrounding medium can possibly change the position, shape and intensity of its absorption and emission spectra. The fluorescence signal of fluorescein also strongly depends on pH, and almost totally vanishes at low acidic pH values which are found in endosomes/lysosomes. Therefore, to develop a reliable method for quantifying intracellular peptide concentrations, cells have to be lyzed in a way that provides homogenous measurement conditions (with disruption of the membrane of all intracellular organelles). Importantly, the lysis conditions have to be compatible with fluorometry and they must ensure that most of the fluorescein fluorescence signal is recovered to obtain an optimal sensitivity of detection.

**Figure 1:**
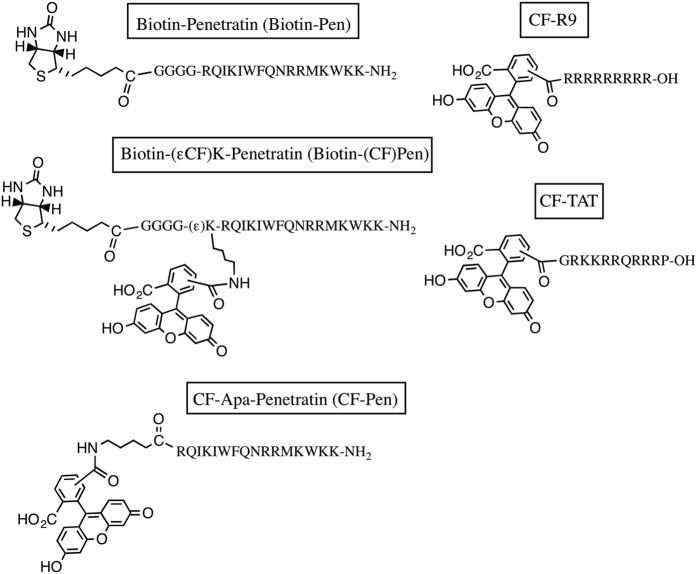
Structure of the peptides used in the study.

**Figure 2:**
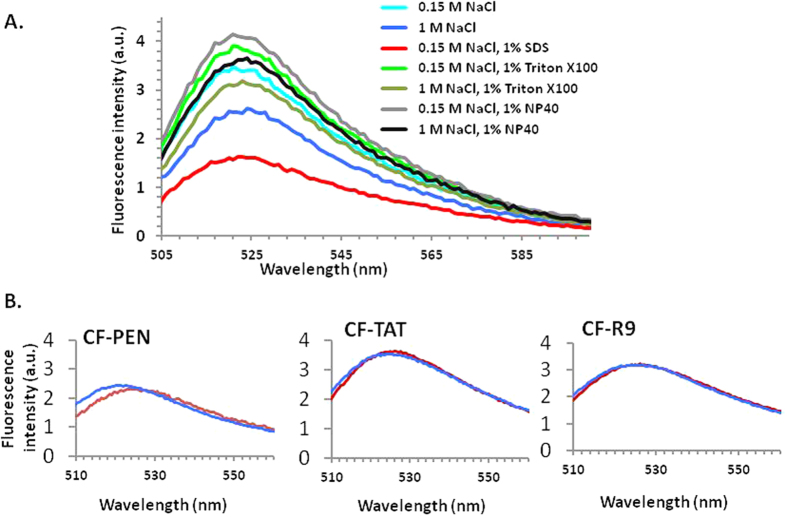
Fluorescence intensity (arbitrary units) in different lysis conditions. (**A**) Variation in fluorescence intensity of 2 μM CF-PEN according to cell (10^6^) lysis conditions. **(B)** 1 μM CF-PEN, CF-R9 or CF-TAT in the optimized lysis buffer (50 mM Tris pH 7.4, 1 M NaCl, 1% Nonidet P40) without cells (red line) and after incubation with cells (blue line).

We first quantified at room temperature and pH 7.4 the fluorescence of the fluorescein-labeled peptide, CF-Penetratin (Fig. 1), in different cell lysis conditions varying in sodium chloride concentration (0.15 M or 1 M) and in type of detergent (1% Triton X-100, sodium dodecylsulfate, or Nonidet P40). CF-Penetratin used at 2 μM, was mixed with cells extracts prepared with different lysis buffers and the fluorescence emission spectra were recorded after excitation at 494 nm (Fig. 2). Disruption of all cell membranes (including membranes of intracellular organelles) was achieved by sonication of cells, in the presence of high NaCl concentration (1 M) to prevent the binding of CPPs to cellular nucleic acids and proteins. The nature of the detergent had a very strong impact on the intensity of the signal in absence of cells. Addition of non ionic detergent indeed improved detection of the fluorescence signal, highest in Nonidet P40 (NP40), decreasing in Triton X-100 and even more in SDS (Fig. 2A). The lysis conditions that gave optimal results with CF-Penetratin (1 M NaCl, 1% NP40, pH 7.4) were then applied to other fluorescein-labeled CPPs (Fig. 2B). The fluorescence emission spectra of CF-Penetratin, CF-TAT and CF-R9 were identical in the lysis buffer alone or mixed with a cell lysate and sonicated. Therefore, the lysis conditions (1 M NaCl, 1% NP40, sonication) were found accurate and reliable, and kept in this study for the measurement of internalized CPPs/PTDs by fluorometry.

### Absolute quantification assays on cell lysates by fluorometry and MALDI-TOF mass spectrometry

**Figure 3:**
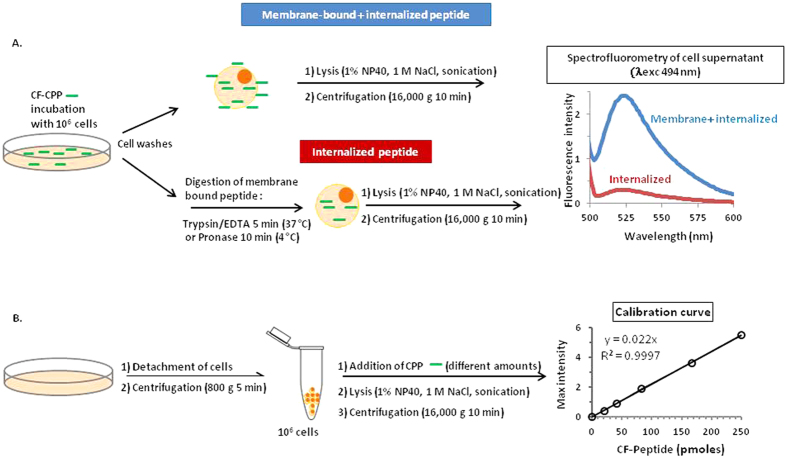
Protocol for CF-CPP quantification by fluorometry. (**A**) Sample preparation and (**B**) calibration sample preparation ran in parallel. See text for protocol description.

To quantify CPP internalization in cells, we kept the conditions of sample preparation for the measurement of the quantity of internalized and cell-associated (membrane-bound plus internalized) fluorescent peptides previously optimized for a MALDI-TOF MS based protocol^14^,^16^, and illustrated in Fig. 3. Briefly, one million adherent cells were incubated with CF-CPP for the indicated time and temperature. Incubation was stopped by several washings before a proteolytic step (with trypsin or pronase when cells had been incubated at 37 °C or 4 °C, respectively) to digest the cell surface bound peptides and to detach cells. Cells were then washed, lysed, sonicated and the lysate was cleared by centrifugation. The amount of internalized peptide was quantified by direct measurement of the fluorescence remaining in the supernatant. To evaluate the quantity of cell-associated (membrane-bound and internalized) peptide, the proteolytic step was omitted. A calibration curve was fitted from the fluorescence intensity of definite amounts of the same peptide in cell lysates ran in parallel for each experiment (Fig. 3). Comparison of the fluorescence intensity between the samples and the calibration curve permitted the absolute quantification of internalized and cell-associated peptides.

**Figure 4:**
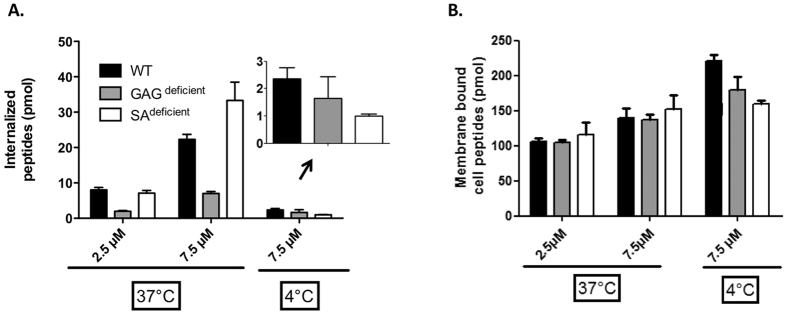
Quantification by fluorometry of (**A**) internalized and (**B**) membrane-bound CF-Penetratin in WT, GAG^deficient^ and SA^deficient^ CHO cells. One million cells were incubated with 2.5 or 7.5 μM CF-Penetratin in DMEM for 60 min at 37 °C or 4 °C. To measure the total cell-associated peptide (membrane-bound plus internalized), cells were washed and lysed in a buffer (50 mM Tris pH 7.4, 1 M NaCl, 1% Nonidet P40), sonicated and centrifuged 10 min at 16,000 g. To obtain the internalized peptide, the membrane-bound peptide was digested with 0.05% trypsin/EDTA (37 °C) or pronase (4 °C). After washes, cells were lysed, sonicated and centrifuged. To calculate the amount of internalized and membrane bound cell peptide, we compare the fluorescence intensity (excitation at 494 nm) to the calibration curve obtained from parallel experiments (see Fig. 3). Error bars correspond to standard errors obtained from n = 4 independent experiments for each experimental condition. Intracellular concentrations (μM) were calculated from the mean volume of one CHO cell (assimilated to a sphere of 10–15 μm diameter) being 1 pL: picomoles in 1 μL (10^6^ cells) thus correspond to μM concentrations.

The protocol was first applied to the study of CF-Penetratin mechanism of entry. We confirmed that the efficacy of CF-Penetratin internalization depended on the temperature and on the cell-surface content in glycosaminoglycans or sialic acid using different CHO lines deficient in glycosaminoglycan (GAG^deficient^) or sialic acid (SA^deficient^) synthesis (Fig. 4, Table 1). At 37 °C and at 7.5 μM the rank of CF-Penetratin internalization efficiency was SA^deficient^ > WT > GAG^deficient^ (Table 1). In contrast at 4 °C, a temperature at which only translocation can occur, similar levels (1–2 μM) of CF-Penetratin were measured in the three cell lines. Quantities of cell-associated CF-Penetratin (membrane-bound plus internalized peptide) were similar in the three cell lines, at both 37 °C and 4 °C (Fig. 4), showing that the peptide that remained associated to the cell membrane after washings, was not bound to GAG or SA sugars.

Regarding Penetratin internalization, fluorometric data were in good agreement with those obtained by MALDI-MS^18^,^19^, although with some differences. When cells were incubated with 7.5 μM Penetratin, the internalization efficacy was found by both techniques to decrease from SA^deficient^ > WT > GAG^deficient^ cells. With fluorometry, we measured higher values of internalized Penetratin than with MALDI-MS, which corresponded to 3.5-, 4.5- and 5.5-fold higher amounts of the peptide in GAG^deficient^, WT and SA^deficient^ cells, respectively. However, because the Penetratin analogues used in the two experiments were not identical: Biotin-GGGG-Penetratin for MS quantification and CF-Apa-Penetratin (Fig. 1) for fluorometry quantification, we wondered whether the different chemical nature of the *N*-terminal sequence in the two Penetratin analogues would interfere with the internalization efficacy.

### Quantification of the same CPP analogue sequence by fluorometry and mass spectrometry

To study whether fluorometry and mass spectrometry actually converge as quantitative analytical methods to measure intracellular concentrations of CPPs/PTDs, we synthesized a bifunctional peptide analogue that could be detected by both techniques. Biotin-G4 and CF tags that allow respectively MS- and fluorometry-based quantification were both introduced on the same peptide molecule (Fig. 1).

**Figure 5:**
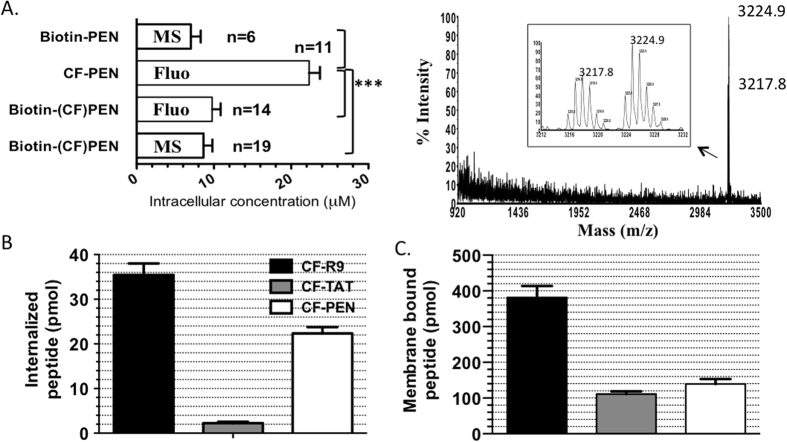
Quantification by fluorometry and/or MALDI-TOF MS. All experiments were done at 37 °C with 7.5 μM extracellular peptide concentration. **(A)** The *left panel* shows the intracellular quantity of Penetratin analogues determined by MALDI-TOF MS (MS) or fluorometry (Fluo). Significance of differences in the means was analyzed with an unpaired t test. The *right panel* shows a typical internalization mass spectrum for Biotin-(CF)Penetratin for which no proteolytic product is observed. The isotopic massif [M + H]^+^ of [^1^H] and [^2^H] Biotin-(CF)Penetratin (corresponding to the internalized peptide and internal standard, respectively) are shown in the inset. **(B)** Intracellular quantity of CF-R9 (black bar), CF-TAT (grey bar) and CF-PEN (white bar) determined by fluorometry. Statistical analysis is given in Supplementary Table S1. **(C)** Quantity of membrane-bound species (for 10^6^ cells) of CF-R9 (black bar), CF-TAT (grey bar) and CF-PEN (white bar). Intracellular concentrations (μM) were calculated from the mean volume of one CHO cell (assimilated to a sphere of 10–15 μm diameter) being 1 pL: picomole amounts in 1 μL (10^6^ cells) thus correspond to μM concentrations.

Keeping the same experimental conditions as before, we determined the intracellular concentration of Biotin-(CF)Penetratin by mass spectrometry and fluorometry (Table 1). Similar values were obtained by both techniques (Table 1, Fig. 5A), and it was deduced from mass spectra that the peptide was not degraded in cells during the one-hour time course (Fig. 5A). Interestingly, measured by MALDI-TOF MS, the intracellular concentrations of the *N*-terminal biotin-labeled peptide containing or not a carboxyfluorescein moiety were found identical (Fig. 5A). By contrast, when measured by fluorometry, the intracellular concentration of CF-Penetratin was significantly increased when the Biotin-G_4_ tag was omitted (4 times, Fig. 5A). Importantly, none of the peptides used in this study was cytotoxic in the concentration and time range tested, as examplified (Supplementary File 1) for CF-Penetratin and Biotin-(CF)Penetratin, ruling out deleterious effects on peptide internalization.

To analyse whether these results could be extended to other CPPs/PTDs, the entry of CF-R9 and CF-TAT into cells was also quantified by fluorometry (Fig. 5B). As for Penetratin, the values of the intracellular concentrations obtained for CF-TAT and CF-R9 by fluorometry were significantly higher (2- and 5-folds for TAT and R9, respectively) than those previously obtained by MS with their biotin-labeled counterparts^14^.

### Relative quantification assays by flow cytometry on intact cells

**Figure 6:**
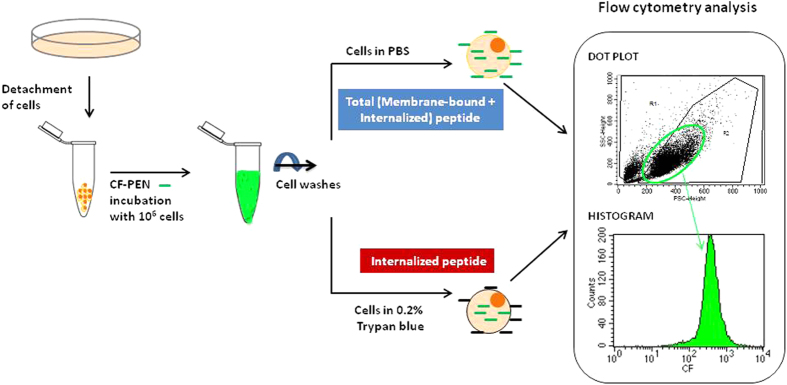
Protocol used for the quantification of total cell-associated (membrane-bound plus internalized) and internalized CF-Penetratin (CF-PEN) by flow cytometry. See text for details of the protocol. In the right inset the dot plot represents the particule size (FSC) versus granularity (SSC) of the cell population. R2 region is choosen to select a homogeneous population of healthy cells. The histogram represents the number of cells from R2 region, displaying a given CF fluorescence signal.

Besides fluorometry and mass spectrometry, flow cytometry is widely used to study cell biomarkers and peptide internalization in cells. The technique has the advantage to quickly give a statistically relevant and quantitative signal of fluorescence with an utmost sensitivity of detection. The general protocol we used is described briefly in Fig. 6.

**Figure 7:**
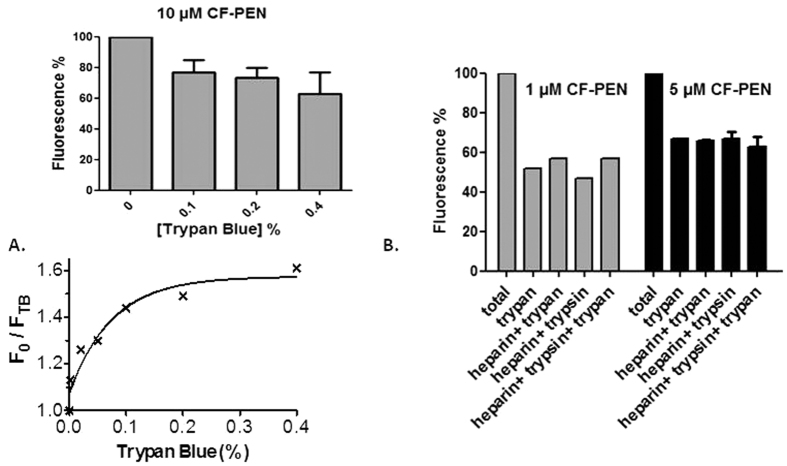
Quenching assays of membrane-bound fluorescence for flow cytometry internalization quantification. (**A**) Fluorescence quenching according to Trypan Blue quantity (% in g for 100 mL). Cells in suspension were incubated 60 min at 37 °C with 10 μM CF-Penetratin. After washing with cold PBS, cells were analyzed in the absence or the presence of Trypan Blue. Lower panel: Stern-Volmer plot. (**B)** Quenching of membrane fluorescence was assayed with various experimental conditions: 0.2% Trypan Blue, 0.01% trypsin (5 min, 37 °C), 0.5 mg/mL heparin (3 times 5 min, 37 °C). 20,000 events were recorded for each experimental condition. Data were normalized to 100% fluorescence in the absence of Trypan Blue.

A major concern, as with all protocols to quantify peptide internalization, was the removal (or quenching) of all membrane-bound species in order to only quantify the internalized peptides. The most common way to quench membrane-bound fluorescein-labelled compounds for flow cytometry analyses is the use of Trypan Blue. Trypan Blue is a non-permeant dye reported to quench the green fluorescence of cell-surface attached particles^19^. We examined the impact of the concentration of Trypan Blue on cell-associated fluorescence compared to control conditions in the absence of Trypan Blue. Cells were incubated with CF-Penetratin. After washings, different concentrations of Trypan Blue were added before cytometry analysis. The fluorescence signal of cell-associated CF-Penetratin was reduced by the addition of increasing concentrations of Trypan Blue (Fig. 7A lower panel). We next examined the quenching ability of Trypan Blue on increasing CF-Penetratin concentrations. We measured the fluorescence signals with (F_TB_) and without (F_0_) Trypan Blue at different CF-Penetratin concentrations (Fig. 7A upper panel and Fig. 7B), and deduced the following F_0_/F_TB_ values: 2 (1 μM), 2.9 (5 μM) and 4 (10 μM). Increasing the extracellular concentrations of CF-Penetratin led to increased amounts of intracellular peptide species (higher F_0_), and as a consequence, to decreased accessibility of Trypan Blue to carboxyfluorescein and an increase of F_0_/F_TB_ ratio.

A number of processes can reduce or quench the intensity of fluorescence. In biological systems, the two quenching processes that are generally encountered are dynamic (collisional) and static (complex formation) quenching. The ratio F_0_/F_TB_ obtained for CF-Penetratin associated to cells, was fitted according to the concentration of Trypan Blue (Fig. 7A). The description for such quenching process stands in the Stern-Volmer derivation equation: F_0_/F_TB_ = 1 + K_SV_ [Q], in which K_SV_ is the Stern-Volmer quenching constant and [Q] the concentration of the quencher (here Trypan Blue). This non-linear Stern-Volmer plot of quenching suggested a dynamic process in which some CF-Penetratin molecules were less accessible than others to the quencher Trypan Blue. These results suggest that the CF-Penetratin inaccessible to Trypan Blue was inside cells. However, as Penetratin is known to insert at least partially into the membrane lipid bilayer^20^,^21^, we further challenged the contribution of cell-surface bound CF-Penetratin to the fluorescence signal measured in the presence of Trypan Blue. To this aim, we tested additional treatments (Fig. 7B) to remove the cell-surface bound peptide: anionic heparin polymer that acts as an electrostatic sponge for the cationic CF-Penetratin, and trypsin protease that cleaves CF-Penetratin sequence at arginyl and lysyl residues. As a similar decrease in the fluorescence signal was reached when the Trypan Blue quenching step was replaced by, or combined with trypsin or heparin treatment, we concluded that all cell-surface associated peptide was sensitive to Trypan Blue treatment alone and consequently, that the fraction of CF-peptide fluorescence unattainable by any of these different treatments (electrostatic sponge, fluorescein quenching, peptide proteolysis) actually corresponded to the intracellular CF-Penetratin signal.

**Figure 8:**
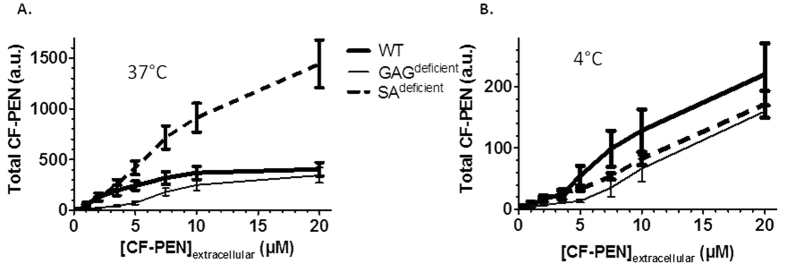
Relative quantification of cell-associated CF-Penetratin (total CF-PEN, membrane-bound plus internalized) by flow cytometry (**A**) at 37 °C and (**B**) at 4 °C in WT, GAG^deficient^ and SA^deficient^ CHO cells. Cells were collected with 0.5 mM EDTA in PBS and suspended with DMEM culture medium. One million cells were incubated with CF-Penetratin in 1 mL DMEM for 60 min. After washes with PBS, cells were analyzed in PBS to measure the signal corresponding to the membrane-bound and internalized peptide. Each sample was analyzed for 20,000 events (BD FACSCalibur). The mean fluorescence values were obtained by subtracting the mean fluorescence of cells without peptide from the measured mean fluorescence in the presence of CF-Penetratin. Error bars are standard deviations obtained from 4 independent experiments for each experimental condition. For statistical analysis, see Supplementary Table S2.

To evaluate peptide association to cells (membrane-bound plus intracellular), Trypan Blue treatment was omitted and cells were only washed with medium following their incubation with different concentrations of CF-Penetratin (Fig. 8). At 37 °C, the fluorescence signal corresponding to the cell-associated peptide, increased linearly with the extracellular concentrations of CF-Penetratin in SA^deficient^ cells, but not with the two other cell lines. An inflexion was observed in the fluorescence signal at extracellular concentration of CF-Penetratin above 3.5 μM for WT cells and 7.5 μM for GAG^deficient^ at 37 °C (Fig. 8). At 4 °C the fluorescence signal of CF-Penetratin peptide kept increasing linearly within the range of concentrations tested, whatever the cell type (Fig. 8). At this temperature, the similar levels of fluorescence signal measured in the three cell lines for each peptide concentration clearly showed that membrane association of CF-Penetratin was mostly independent of the cell-surface carbohydrate composition.

**Figure 9:**
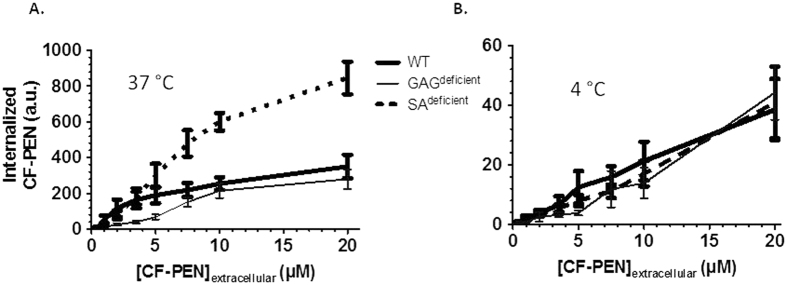
Internalized CF-Penetratin relative quantification by flow cytometry (**A**) at 37 °C and (**B**) at 4 °C, in WT, GAG^deficient^ and SA^deficient^ CHO cells. Similar experimental conditions as described in the legend of Fig. 8, except that cells were analyzed in 0.2% Trypan Blue in PBS to measure the internalized peptide. For statistical analysis, see Supplementary Table S3.

In a second set of experiments, cells were treated with Trypan Blue to quench the extracellular CF-Penetratin and to measure only intracellular CF-Penetratin signal in WT, GAG^deficient^ and SA^deficient^ cells (protocol described in Fig. 6). At 37 °C (Fig. 9), a proportional increase of the fluorescence signal with external CF-Penetratin concentration occurred in SA^deficient^ cells up to 10 μM, but reached a plateau at concentrations greater than 5 μM CF-Penetratin for WT and 10 μM for GAG^deficient^.

At 4 °C, a temperature at which all endocytosis processes are inhibited and therefore only translocation can occur, albeit with lower efficiency due to decreases in cell membrane fluidity and dynamics, there was no difference in the amount of internalized CF-Penetratin between the three cell lines, whatever the concentration, but internalization levels were lower (15- to 20-folds) compared to 37 °C. It indicates that at this temperature the cell-surface content in carbohydrates is not influencing internalization, whatever the peptide concentration.

At 37 °C, at concentrations above 5 μM, the similar amounts of CF-Penetratin in WT and GAG^deficient^ cells (Fig. 9) suggested the occurrence of fluorescence signal quenching. We have indeed previously reported by mass spectrometry a difference between WT and GAG^deficient^ cells in the internalization efficacy of Penetratin, at least up to 30 μM of extracellular peptide^17^,^22^.

### Evidence for fluorescence quenching in intact cells

**Figure 10:**
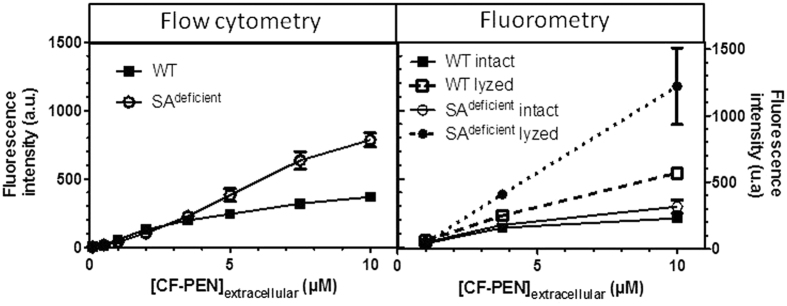
Internalized CF-Penetratin relative quantification in WT and SA^deficient^ cells by flow cytometry or fluorometry. Cells were incubated with CF-PEN and prepared according to the protocol for flow cytometry described in Fig. 8. Fluorescence in intact cells was measured by flow cytometry or fluorometry (3 independent experiments). Cells were then lyzed to release all fluorescence peptide and detect quenching (3 independent experiments) by fluorometry.

To test the hypothesis that the fluorescence signal might be quenched in intact cells, we analyzed by fluorometry cells prepared with the flow cytometry protocol in the absence of Trypan Blue, to allow detection of membrane-bound and internalized fluorescent species. Fluorescence was measured either on intact cells or cell lysates (Fig. 10). Experiments were done with WT and SA^deficient^ cells that showed differences in inflections of the fluorescence signals measured by flow cytometry (Fig. 8): an inflection of the fluorescence signal was indeed reached with WT cells for concentrations of CF-Penetratin above 3.5 μM but not for SA^deficient^ cells up to 10 μM. As shown in Fig. 10, the fluorescence signal measured by fluorometry was higher in cell lysates than in intact cells, showing that quenching of fluorescence occurred in both WT and SA^deficient^ intact cells. The extent of quenching clearly depended on CF-Penetratin concentration and was surprisingly higher for SA^deficient^ cells than for WT cells. For instance at 3.5 μM CF-Penetratin, an identical level of peptide fluorescence signal was measured in SA^deficient^ and WT intact cells. However once lyzed, the fluorescence signal of CF-Penetratin was higher for SA^deficient^ cells than for WT cells. Interestingly, we measured similar fluorescence in lysates and intact cells at 1 μM CF-Penetratin for the two cell lines, showing the absence of quenching at this low μM concentration. At 10 μM CF-Penetratin, 2.5 and 3.8 folds higher fluorescence were measured in lysates versus intact cells for WT and SA^deficient^, respectively.

Altogether, data from flow cytometry showed that: i) at 37 °C, internalization efficiency of CF-Penetratin appeared to depend on the composition of cell membrane in negatively charged sulfated glycosaminoglycans and sialic acids; however, above a threshold concentration of CF-Penetratin, quenching of the fluorescence signal occurred to different degrees depending on the nature of the cell line, leading consequently to distortion in the interpretation of the data; ii) at 4 °C, translocation was independent of the anionic cell-surface components.

## Discussion

Most studies devoted to the internalization efficacy of CPPs/PTDs rely on the use of fluorescence techniques, microscopy imaging, fluorometry or flow cytometry, to detect and track cell-penetrating peptides in cells. A controversy on CPPs entry mechanisms arose in the literature because these peptides have strong membranotropic activity. Therefore, the study of their internalization efficacy by fluorescence techniques requires accurate, robust and efficient sample preparations and protocols. We had previously reported a method involving MALDI-TOF mass spectrometry to measure absolute concentrations of cell-penetrating peptides inside cells^14^. This protocol also permitted to quantify the membrane-bound peptide species and detect the degradation of CPPs/PTDs inside cells^14^. The major drawbacks of this MS protocol is that it requires: i) the synthesis of both ^1^H and ^2^H isotopic forms of the biotin-tagged peptide sequence, in order to allow quantification using the heavy deuterium-labeled peptide as an internal standard in mass spectra, and ii) an access to a MALDI-TOF equipment.

In this context, we wanted herein to evaluate whether a similar way of sample preparation could be suitable to quantification by fluorometry and to test whether fluorometry and mass spectrometry would converge on the quantity of cell-penetrating peptides measured in cells. Such comparative study had never been performed and is critical to validate a general way of sample preparation for absolute quantification of internalized peptides, in particular with regard to the elimination of membrane-bound species.

We chose Penetratin CPP sequence and carboxyfluorescein fluorochrome because both are widely studied and used. The protocol was adapted from our previous MALDI-TOF MS experiments, and roughly involves a proteolytic degradation step of the membrane-bound peptide species prior to cell lysis in high salt (1 M NaCl) and detergent (Nonidet P40) to disrupt and solubilize cell membranes, and prevent binding of the peptides to intracellular molecules such as proteins or DNA. Using this protocol, we considered that the peptide inacessible to trypsin at 37 °C or pronase at 4 °C, is inside cells, as observed previously by confocal microscopy^15^,^23^ or measured by mass spectrometry^15^,^23^,^24^. A sonication step was added to ensure the complete solubilization of intracellular organelle membranes. We found that this protocol performed at controlled pH 7.4, permitted to measure identical quantity of internalized Biotin-(CF)Penetratin peptide with both techniques, fluorometry and mass spectrometry. Identical quantities were also measured for Biotin-(CF)Penetratin and Biotin-Penetratin by mass spectrometry. Strikingly, for the CF-Penetratin analogue, for which CF was moved from a lysyl side-chain to the peptide *N*-terminus (to replace the biotin-(Gly_4_) tag), a strong increase in the internalization of Penetratin was observed. Replacement of the *N*-terminal biotin-(Gly_4_) by a more hydrophobic carboxyfluorescein moiety appeared to have a positive influence on the internalization efficiency of the three well studied CPPs Penetratin, TAT and R9 (from 2 to 5 times). A similar effect has already been reported with other modifications. Single Gly to Phe change at the *N*-terminus of a neutral bridging sequence, upstream of a cargo linked to a CPP, significantly enhanced the vector properties of the CPP^25^. It suggests that the position of the fluorochrome has a strong impact on the internalization efficacy or alternatively, that the *N*-terminal biotin-(Gly_4_) tag is detrimental to the internalization efficiency of the CPP. In both cases, it indicates that the hydrophic/hydrophilic property of the *N*-terminal ending group in the peptide sequence is crucial for CPP/PTD efficacy. Whether this finding that applied to Penetratin, TAT and R9 is a general rule for all CPPs/PTDs is an interesting question that needs to be further studied.

In addition, the results of quantification by fluorometry also confirmed the importance of cell-surface glycosaminoglycans in the internalization efficacy of Penetratin, at least at some CPP concentrations. Indeed, a significant decrease in Penetratin internalization was obtained with GAG^deficient^ cells, an observation previously reported for many CPPs/PTDs^15^,^26^,^27^,^28^,^29^,^30^. A significant increase in Penetratin internalization was also observed with SA^deficient^ cells, an observation that was also previously reported using mass spectrometry as the quantification method^17^. After one hour incubation with 7.5 μM extracellular Penetratin, 22 μM are indeed found in WT cells and 35 μM are measured in SA^deficient^ cells. Thus, Penetratin ended up to be more concentrated inside cells than outside, meaning that the peptide accumulated in cells during the time course of the experiment. This demonstrates that Penetratin internalization into cells does not (only) rely on a diffusion process and that the peptide recruits cell surface partners to go into cells. In addition, internalization is more efficient in SA^deficient^ cells compared to WT cells which may be explained by the fact that in WT cells, SA trap Penetratin at the cell surface competing/preventing its interaction with membrane components involved in internalization, as we previously proposed^17^,^18^.

Altogether these results indicate that fluorometry is a good alternative to mass spectrometry for the absolute quantification of CPPs/PTDs. However, contrary to MS, fluorometry does not allow to distinguish between intact and degraded internalized CPP and to analyze the metabolic fate of peptides within cells, techniques such as ultra fast and high separation liquid chromatography are then required^31^. Mass spectrometry and fluorometry-based methods give complementary information: the amount of intact internalized CPP is measured by MS, whereas fluorometry gives access to the total amount of internalized peptide (including intact and potential degraded species). The Penetratin bifunctional analog (Biotin(CF)Pen) studied herein was not quickly degraded inside cells and similar amounts of intracellar peptide were measured by both technics after one hour incubation.

Besides fluorometry, flow cytometry is frequently used to study internalization of fluorescent CPPs/PTDs. In this case, cells are intact when analyzed. The use of intact cells might bring a bias in the interpretation of flow cytometry data because of potential quenching phenomenon occurring in cells, as we evidenced herein. For instance, it is well known that in nonpolar or low pH environments the fluorescein chromophore might exist under nonfluorescent chemical forms. The evidence for aggregation and formation of at least two different fluorescein-conjugate aggregated species, one of them being fluorescent while the other was not, was brought for example by the spectroscopic analysis of (5-n-hexadecanoylamino)-fluorescein mixed with palmitic acid in Langmuir-Blodgett films^32^. In addition, as a membranotropic peptide, Penetratin can insert at least partially into phospholipid membrane vesicles^20^,^21^. Therefore non-fluorescent CF-Penetratin species exist in cell plasma membrane depending on peptide environment. A similar situation can occur inside cells, provided that a concentration threshold is reached in confined regions of the cell leading to fluorescence self-quenching as we demonstrated herein, which deleterious effect on the fluorescent signal can be further amplified in acidic compartments such as endosomes. This kind of quenching was also recently reported for confocal laser scanning microscopy experiments^33^. Quenching might also contribute to the small difference (1.25 to 2 folds) observed between the total cell-associated and the internalized fluorescence signals measured by flow cytometry for penetratin, contrasting with the ratio obtained by fluorometry and mass spectrometry, close to 100 folds for this peptide. The peptide/cell ratio has been described as a relevant parameter to describe CPP uptake^34^. Coherently, it appears to be also relevant to describe the threshold of quenching of the fluorescent probe conjugated to the peptide. In the present study we have used peptide/cell ratio from 6 × 10^7^ (0.1 μM peptide incubated in 1 mL with 10^6^ cells) to 1.2 × 10^10^ (20 μM peptide incubated in the same conditions). In these conditions, 1 μM peptide concentration corresponds to peptide/cell ratio of 6 × 10^8^, for which no quenching was observed. Our results also highlight the difference in the extent of quenching according to the cell line, showing that quenched fluorescent species strongly rely on cell-surface components, thus also on internalization pathways for intracellular quenched species.

The formation of non-fluorescent aggregates also explains the fall of the fluorescence signal according to CF-Penetratin concentration that we observed at 37 °C in flow cytometry but not in fluorometry. Only in the latter case, the CF-peptide fluorescence is released from confined organelles or regions of the cell before measurements, and thus fully recovered and measured. In addition, when the fluorometry method is used, the heterogenous environment (polar/apolar, acidic/neutral) of the cell interior is homogenized and buffered at neutral pH. In parallel, calibration curves were performed with different amounts of the appropriate CF-CPP added to cell lysates prepared in the same experimental conditions.

In this context, our results clearly show that for comparative and quantitative studies of peptide inside intact cells, one should be cautious with the peptide concentrations and experimental conditions to be used. In flow cytometry, we show here that the fluorescence signal from the internalized peptide in WT and GAG^deficient^ cells, no longer increased for CF-Penetratin concentrations above 5 μM. By contrast, when measured by fluorometry or mass spectrometry, the relative quantities of the internalized peptide in WT and GAG^deficient^ cells were significantly different between the two cell lines at concentrations of Penetratin above 5 μM. Thus, depending on the experimental conditions used, differences in CF-CPP internalization between cell lines can be masked when using flow cytometry. This phenomenon might account, at least partly, for conflicting reports in the literature regarding the role of glycosaminoglycans in the internalization of CPPs.

In conclusion, absolute quantification of internalization pathways of CPPs/PTDs is possible by fluorometry and mass spectrometry and both techniques converge, as tested herein for different cell lines and peptide sequences. Relative quantification data by flow cytometry also merge with those obtained by fluorometry and mass spectrometry, but in a limited range of peptide/cell ratio. The protocols used for mass spectrometry and fluorometry techniques are destructive for cells but allow the accurate detection of the full peptide signal (absence of quenching, controlled pH = 7.4), when flow cytometry, a non-intrusive technique like fluorescence imaging, is limited by the cell structure and organization and local pH value that interfere with and distort the recovery of the full fluorescence signal. Therefore, and whatever the fluorescence technique used to perform quantitative analyses, one should be cautious and test different peptide/cell ratios, ensure the complete removal of membrane-bound peptide species, and take into account quenching processes and the possible formation of non-fluorescent peptide species (formation of aggregates, acidic pH), which both introduced distortion in the measurements and therefore in their interpretation. Our study also pointed out that the hydrophilic/hydrophobic nature of the chemical moiety positioned at the *N*-terminal extremity of the peptide sequence is critical to the internalization efficiency of the corresponding peptide. Finally, our data confirmed that below 2 μM concentration (peptide/cell ratio below 1.2 × 10^9^), Penetratin translocates, and that this peptide uses additional endocytic pathways at higher concentrations^16^,^35^.

Altogether, we hope that the accurate use of these complementary techniques will be helpful to decipher the internalization pathways and traffick of CPPs/PTDs or cargo molecules, and to further design efficient peptide vectors for delivery in the cytosol of cells. We expect that these results and the associated protocols will help unraveling the translocation pathway to the cytosol of cells. More generally this study should be of interest for any quantitative study of fluorescent molecules in cells, beyond the field of CPPs/PTDs.

## Materials and Methods

### Peptides

Peptides were obtained by solid-phase synthesis using the Boc strategy, except for FAM-R9 (CF-R9) and FAM-TAT (CF-TAT) that were purchased from Eurogentec. Peptides (Fig. 1) were all purified by RP-HPLC and further checked by mass spectrometry: Carboxyfluorescein-Apa-RQIKIWFQNRRMKWKK-NH_2_ (CF-Penetratin) MW = 2700; Biotin-GGGGRQIKIWFQNRRMKWKK-NH_2_ (Biotin-Penetratin), MW = 2699 (^1^H-Gly) and MW = 2707 (^2^H-Gly); Biotin-GGGG(εCF)KRQIKIWFQNRRMKWKK-NH_2_ (Biotin-(CF)Penetratin), MW = 3216 (^1^H-Gly) and MW = 3224 (^2^H-Gly). Carboxyfluorescein-RRRRRRRRR-OH (CF-R9), MW = 1782; Carboxyfluorescein-GRKKRRQRRRP-OH (CF-TAT), MW = 1918. Concentration of stock solutions were obtained from weighted peptides, taking into account the mass of trifluoroacetate (TFA) counteranions, and checked by NMR.

### Cell Culture

Wild type Chinese Hamster Ovary CHO-K1 cells (WT), xylose-transferase- or GAG-deficient CHO-pgsA745 cells (GAG^deficient^) and sialic acid deficient CHO-lec2 cells (SA^deficient^) (ATCC, LGC Standards S.a.r.l. - France) were cultured in F12 growth medium (DMEM-F12) supplemented with 10% fetal calf serum (FCS), penicillin (100,000 IU/L), streptomycin (100,000 IU/L), and amphotericin B (1 mg/L) in a humidified atmosphere containing 5% CO_2_ at 37 °C.

### Cytotoxicity Assays

Cytotoxicity was determined with the Cell Counting Kit 8 (CCK-8) from Dojindo Molecular Technologies. This colorimetric assay allows to measure the viability of cells. 96-well plates were inoculated with 100 μL/well of a suspension of CHO cells (2.10^4^ cells/well). After 24 h of incubation (37 °C, 5% CO_2_) 10 μL of peptide (0; 1; 5; 10 and 20 μM final) were added and the plate was further incubated for 60 min. After washing, the cells were incubated with 100 μL of 10% CCK-8 in DMEM for 3 hours and the absorbance was measured at 450 nm with a microplate reader (Polarstar Optima). Controls corresponded to untreated cells (negative control, 100% viability) and cells treated with 0.2% of Triton X-100 (positive control, 0% viability).

### Flow cytometry quantification assays

#### Quenching studies

Cells were dissociated with 0.5 mM EDTA in PBS (5 min at 37 °C), centrifuged at 800 g, and suspended in DMEM culture medium. One million cells in suspension were incubated for 60 min at 37 °C with or without peptide (10 μM in 1 mL DMEM). Cells were centrifuged at 800 g at 4 °C. The cell pellets were washed with cold PBS, centrifuged at 800 g at 4 °C, and supended in PBS. To optimize fluorescence quenching of the membrane-bound fluorescein, the cell suspension was treated with different concentrations of Trypan Blue. The fluorescence of individual cells was measured by flow cytometry using a FACSCalibur (BD Biosciences) and analyzed by the BD CellQuest^TM^ software. Wavelengths were 488 nm for excitation and 525 nm for detection. 20,000 events were recorded for every experimental conditions. To determine the best fluorescein quenching conditions, we incubated cells in the absence or the presence of (1 μM or 5 μM) peptide for 60 min at 37 °C, and we compared different cell treatments, combining up to the three following compounds: 0.2% Trypan Blue, 0.01% trypsin for 10 min at 37 °C, 0.5 mg/mL heparin (3 times 5 min) at 37 °C.

#### Quantification of internalization by flow cytometry

To analyze the internalization of fluorescein-labeled Penetratin peptides by flow cytometry, cells were dissociated with 0.5 mM EDTA in PBS (5 min at 37 °C), centrifuged at 800 g, and suspended in DMEM culture medium. 2.10^6^ cells were incubated (37 °C or 4 °C) with peptides at different concentrations (0.1, 0.5, 1, 2, 3.5, 5, 7.5, 10 and 20 μM in 1 mL DMEM). After incubation, cells were centrifuged at 800 g at 4 °C. The cell pellet was washed with cold PBS, centrifuged at 800 g and suspended in 400 μL of PBS.

Half of the cell suspension (one million cells in 200 μL) was used to measure the total fluorescence (membrane-bound and internalized peptide). The other half was treated with 0.2% Trypan Blue (in PBS) to quench the membrane-bound fluorescence and access the internalized peptide. The fluorescence of individual cells was analyzed with a FACSCalibur flow cytometer. 20,000 cells were measured for each experimental condition. The mean fluorescence of a sample was obtained by subtracting the autofluorescence of cells from the measured mean fluorescence of cells in the presence of the fluorescent peptide.

### Quantification assays by fluorometry

#### Lysis conditions

Various detergents and ionic strength conditions were tested to optimize cell lysis solution and find conditions that would allow full fluorescence recovery for the quantification measurements. CHO cells were detached with (0.05%) trypsin/EDTA, counted and splitted to obtain 1 million cells suspended in 100 μL DMEM per microtube. After centrifugation at 800 g, different lysis solutions at room temperature (200 μL) were added to each microtube containing or not 2 μM CF-Penetratin: 50 mM Tris pH 7.4, 0.15 M or 1 M NaCl, 1% Nonidet P40 or SDS or Triton X-100. The control experiments were done in the absence of cells. Samples were sonicated for 30 min and centrifuged 10 min at 16,000 g. Fluorescence in the supernatants was analyzed with a MOS 200 M fluorimeter (Biologic SAS, France), an excitation wavelength at 494 nm and emission was monitored between 500 and 600 nm with an acquisition duration of 0.2 sec/0.5 nm.

#### Quantification assays

One million cells were incubated one hour at 37 °C or 4 °C with CF-Penetratin (2.5 μM and 7.5 μM), Biotin-(CF)Penetratin, CF-Arg9 and CF-TAT (7.5 μM) in 1 mL DMEM.

To access internalized peptides, after washing cells with HBSS, (0.05%) 500 μL trypsin/EDTA (37 °C, 5 min) or (0.05%) pronase (4 °C, 10 min) was added for 5 min to hydrolyze the remaining extracellular and the membrane-bound peptide and to detach cells. After addition of enzyme inhibitors (100 μL (one Complete Mini tablet, Roche, in 2.5 mL PBS) mixed with 100 μL bovine serum albumin (1 mg/mL), cells were transferred into a microtube, centrifuged, washed with 1 mL 50 mM Tris buffer pH 7.4, 0.1% BSA, and lysed in 200 μL 50 mM Tris pH 7.4, 1 M NaCl, 1% NP40. The samples were then sonicated for 30 min and centrifuged 10 min at 16,000 g.

To obtain the value of the total cell-associated peptide (internalized and membrane-bound species), after washing cells with HBSS, cells were directly lysed in 50 mM Tris pH 7.4, 1 M NaCl, 1% NP40. The samples were then sonicated for 30 min and centrifuged 10 min at 16,000 g. Fluorescence intensity in the supernatants was monitored with a MOS 200 M fluorimeter (Biologic SAS, France) between 500 and 600 nm (0.2 sec/0.5 nm), and the maximal intensity was detected around 520 nm. The maximal intensity around 520 nm was retained for the calibration curve and for quantification of samples. The amounts of total or internalized peptide were calculated by comparing the fluorescence intensity of the sample with the calibration curve (illustrated in Fig. 2). Samples for the calibration curve were prepared in parrallel. For this, 10 different amounts (from 2 to 500 pmoles) of the appropriate CF-CPP were added to one million cells suspended in 200 μL lysis buffer. As indicated in the figures, all experiments were perfomed in duplicates and repeated at least twice independently.

Calculation of the amount of internalized and non internalized peptide was obtained from comparison of the fluorescence intensities of the samples and a calibration curve (Fig. 3). In parallel with the samples, we prepared a range of peptide amounts in the lysis buffer (50 mM Tris pH 7.4, 1 M NaCl, 1% Nonidet P40) in the presence of one million suspended cells. The samples were sonicated 30 min and centrifuged at 16,000 g for 10 min. Fluorescence was then measured in supernatants. The amounts of total or internalized peptide were calculated by comparing the fluorescence intensity of the sample with the calibration curve. For each experimental condition we used duplicate wells, and the experiments were all repeated independently at least two times, as indicated.

### Quantification of peptides by mass spectrometry

The protocol has been fully described elsewhere by Burlina *et al.*^14^,^16^. Briefly, one million adherent cells were incubated one hour with biotin-labeled peptides (^1^H-CPP). After washing with HBSS, 0.05% trypsin/EDTA (37 °C, 5 min) or 0.05% pronase (4 °C, 10 min) digestion, cells were lysed in a solution containing a relevant quantity of the deuterated form (^2^H-CPP) of the same peptide sequence, which is used as internal standard to quantify the internalized amount of ^1^H-CPP. MALDI-TOF MS analyses were done using cyano-4-hydroxy-cinnamic acid (CHCA), for Biotin-Penetratin or sinapinic acid for Biotin-(CF)Penetratin. Unless indicated, experiments were performed in triplicates and repeated at least three times independently.

## Additional Information

**How to cite this article**: Illien, F. *et al.* Quantitative fluorescence spectroscopy and flow cytometry analyses of cell -penetrating peptides internalization pathways: optimization, pitfalls, comparison with mass spectrometry quantification. *Sci. Rep.* **6**, 36938; doi: 10.1038/srep36938 (2016).

**Publisher’s note**: Springer Nature remains neutral with regard to jurisdictional claims in published maps and institutional affiliations.

## Supplementary Material

Supplementary Information

## Figures and Tables

**Table 1:** Quantitative data for intracellular Penetratin analogues, determined by MALDI-TOF MS and fluorometry.

MALDI-TOF MS	37 °C - INTERNALIZED (μM)			4 °C - INTERNALIZED (μM)		
FLUOROMETRY	WT	GAG ^deficient^	SA ^deficient^	WT	GAG ^deficient^	SA ^deficient^
Biotin-PEN^19^	5.0 ± 0.5	1.9 ± 0.2	6.0 ± 1.0	0.9 ± 0.2	0.6 ± 0.1	1.0 ± 0.2
Biotin-(CF)PEN	8.7 ± 1.1 (n = 19)	*Not detectable*	—	*Not detectable*	—	—
**CF-PEN**	**22** ± **1.3 (n** = **11)**	**7.0** ± **0.5 (n** = **4)**	**33** ± **5.3 (n** = **4)**	**2.4** ± **0.4 (n** = **3)**	**1.7** ± **0.8 (n** = **3)**	**1.0** ± **0.1 (n** = **4)**
**Biotin-(CF)PEN**	**9.8 ±1.0 (n** = **14)**	**1.1** ± **0.1 (n** = **4)**	**6.9** ± **1.8 (n** = **3)**	**1.5** ± **0.1 (n** = **10)**	**—**	**—**

Data obtained by MALDI-TOF MS for Biotin-PEN were previously reported in ref. 19 and added for comparison. Statistical analysis is given in Supplementary Table S1.
