# Supplementary material for: Quantitative fluorescence spectroscopy and flow cytometry analyses of cell-penetrating peptides internalization pathways: optimization, pitfalls, comparison with mass spectrometry quantification
Source: Sci Rep. 2016 Nov 14;6:36938. doi: 10.1038/srep36938 (PMC5107916; doi:10.1038/srep36938)
Supplement: Supplementary Information [file srep36938-s1.doc]

**SUPPLEMENTARY DATA**

**Quantitative fluorescence spectroscopy and flow cytometry analyses of cell-penetrating peptides internalization pathways: optimization, pitfalls, comparison with mass spectrometry quantification**

Françoise Illien^1,2^, Nicolas Rodriguez^1,2^, Mehdi Amoura^1,2^, Alain Joliot^3^, Manjula Pallerla^1,2^, Sophie Cribier^1,2^, Fabienne Burlina^1,2^, Sandrine Sagan^1,2 *^

*^1^ Sorbonne Universités, UPMC Univ Paris 06, Ecole Normale Supérieure, CNRS, Laboratoire des Biomolécules (LBM), 4 place Jussieu, 75005 Paris, France.*

*^2^ Département de Chimie, Ecole Normale Supérieure, PSL Research University, UPMC Univ Paris 06, CNRS, Laboratoire des Biomolécules (LBM), Paris, France.*

*^3^ Center for Interdisciplinary Research in Biology, Collège-de-France, PSL Research University, 11 place M. Berthelot, Paris, France.*

* Correspondence to S. Sagan

Université Pierre and Marie Curie

Laboratoire des Biomolecules, CC 182

4, place Jussieu

75252 Paris cedex 05, France

Tel. +33 1 44 27 61 99

sandrine.sagan@upmc.fr

sandrine.sagan@ens.fr

**
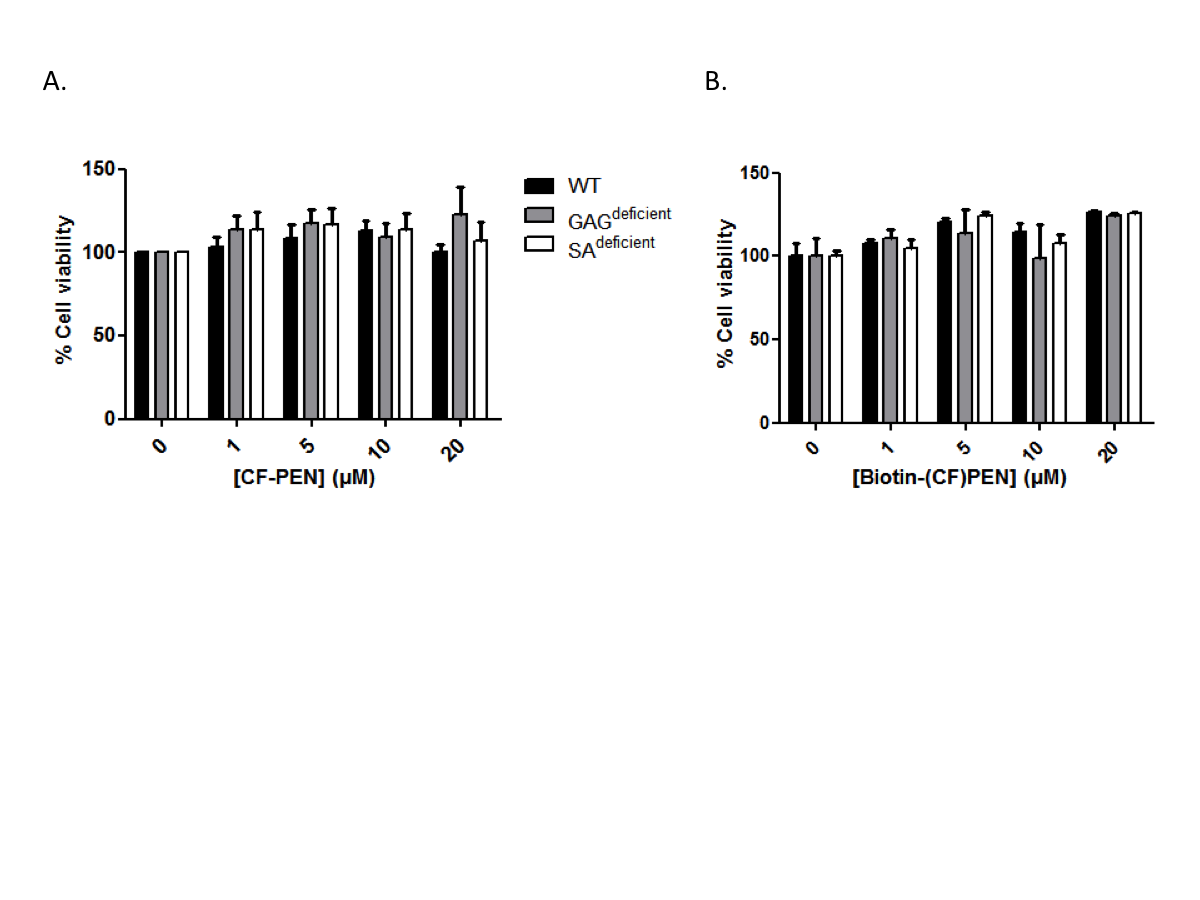
**

**Supplementary Figure S1. Cytotoxicity assays of CF-Penetratin (A) and biotin-(CF) Penetratin (B)** were determinated by the Cell Counting Kit 8 (CCK8), a colorimetric assay in which the absorbance is proportional to the number of living cells. A 96-well plate was inoculated with 100 μL/well of a suspension of CHO cells (2 × 10^4^ cells/well). After 24 h of incubation (37°C, 5% CO_2_) 10 μL of peptide (0; 1 ; 5; 10 and 20 µM final) were added for 1 hour. After removing the peptide solution, the cells were further incubated for 3 h with 100 μL of 10% CCK-8 solution in DMEM, before the absorbance was measured at 450 nm. Controls corresponded to untreated cells (negative control, 100% viability) and cells treated with 0.2% of Triton X-100 (positive control, 0% viability).

**Supplementary Table S1 – Comparison for significant differences in Penetratin internalized quantity obtained at 37°C by flow cytometry (CF-Penetratin), fluorometry (CF-Penetratin) and mass spectrometry (Biotin-G_4_-Penetratin) assays.** Pairs of columns were compared using unpaired t test. Differences were ranged from not significant (ns) to extremely significant (***), as follows : ns, p≥0.05, * 0.01<p<0.05; ** 0.001<p<0.01; ***p<0.001. ; (-) not determined.

| **Penetratin**  **Concentrations (μM)** | **0.1** | **0.5** | **1** | **2** | **3.5** | **5** | **7.5** | **10** | **20** |
| --- | --- | --- | --- | --- | --- | --- | --- | --- | --- |
|  | **WT *versus* GAG^deficient^** | | | | | | | | |
| Flow Cytometry | * | ns | ns | ns | * | * | ns | ns | ns |
| Fluorometry | - | - | - | *** | - | - | *** | - | - |
| Mass Spectrometry | ns | ns | ns | * | * | ** | ** | ** | - |
|  | **WT *versus* SA^deficient^** | | | | | | | | |
| Flow Cytometry | ns | ns | ns | ns | ns | ns | * | ** | ** |
| Fluorometry | - | - | - | ns | - | - | * | - | - |
| Mass Spectrometry | ns | ns | ns | ns | ns | * | * | ** | - |
|  | **GAG^deficient^ *versus* SA^deficient^** | | | | | | | | |
| Flow Cytometry | ns | ns | * | * | ** | ** | ** | ** | ** |
| Fluorometry | - | - | - | *** | - | - | ** | - | - |
| Mass Spectrometry | ns | ns | ns | ns | * | * | ** | ** | - |

**Supplementary Table S2** : **Statistical comparison of means of cell-associated CF-PEN fluorescence intensity determined by flow cytometry at 37°C in WT, GAG^deficient^ and SA^deficient^, according to extracellular peptide concentrations.** Pairs of columns were compared using unpaired t test. ns, not significant p≥0.05; *significant, p<0.05; **very significant, p<0.01; (-) not determined.

| concentration (μM) | 0.1 | 0.5 | 1 | 2 | 3.5 | 5 | 7.5 | 10 | 20 |
| --- | --- | --- | --- | --- | --- | --- | --- | --- | --- |
| WT vs GAG^def^ | ns | ns | ns | ns | * | * | ns | ns | ns |
| WT vs SA^def^ | ns | * | ns | ns | ns | ns | ** | ** | ** |
| GAG^def^ vs SA^def^ | ns | * | * | ** | ** | ** | ** | ** | ** |

**Supplementary Table S3 : Statistical comparison of means of internalized CF-PEN fluorescence intensity determined by flow cytometry at 37°C in WT, GAG^deficient^ and SA^deficient^, according to extracellular peptide concentrations.** Pairs of columns were compared using unpaired t test. ns, not significant p≥0.05; *significant, p<0.05; **very significant, p<0.01; (-) not determined.

| concentration (μM) | 0.1 | 0.5 | 1 | 2 | 3.5 | 5 | 7.5 | 10 | 20 |
| --- | --- | --- | --- | --- | --- | --- | --- | --- | --- |
| WT vs GAG^def^ | * | ns | ns | ns | * | * | ns | ns | ns |
| WT vs SA^def^ | ns | ns | ns | ns | ns | ns | * | ** | ** |
| GAG^def^ vs SA^def^ | ns | ns | * | * | ** | ** | ** | ** | ** |
